# Negative Correlation Between Serum Ferritin and CD4+ Lymphocytes: A Retrospective Study on Kikuchi‐Fujimoto Disease

**DOI:** 10.1002/iid3.70358

**Published:** 2026-02-12

**Authors:** Peng Zhong, Xiwen Sang, Yu Yang, Zhenzhou Wang

**Affiliations:** ^1^ Department of Ultrasound, Beijing Friendship Hospital Capital Medical University Beijing People's Republic of China; ^2^ Medical College of Shanxi Datong University Datong Shanxi People's Republic of China; ^3^ Department of Intensive Care Medicine Trauma Center, Peking University People's Hospital Beijing People's Republic of China

**Keywords:** CD4+ lymphocytes, ferritin, Kikuchi–Fujimoto disease, lymphocyte subtype

## Abstract

**Background:**

Kikuchi–Fujimoto disease (KFD) is a rare condition characterized by elevated serum ferritin levels and a robust immune response involving various lymphocyte subtypes. However, the relationship between ferritin and lymphocyte subtypes remains unexplored. This study aims to offer insights into the immune response in KFD.

**Methods:**

Sixty‐five hospitalized patients diagnosed with KFD through histopathological examination were categorized into hyperferritinemia and control groups. Baseline characteristics, ultrasound findings, and laboratory results were retrospectively collected for analysis.

**Results:**

Biopsied lymph nodes in KFD patients were predominantly located in the neck (89.2%), followed by the axilla (6.2%) and submandible (4.6%). These nodes exhibited a mean long diameter of 2.3 mm (interquartile range [IQR] 2.0–2.8 mm) and a mean short diameter of 0.9 mm (IQR 0.7–1.2 mm). A comparison between the hyperferritinemia and control groups revealed significant differences: Serum ferritin levels were 664.4 µg/L (IQR 450.5–963.9 µg/L) vs. 111.3 µg/L (IQR 75.7–207.4 µg/L), respectively. Additionally, counts of CD3+ and CD4+ lymphocytes, as well as NK cells, were reduced in the hyperferritinemia group: CD4+ lymphocyte counts were 387.7 cells/µL (IQR 261.5–479.7 cells/µL) vs. 479.9 cells/µL (IQR 396.2–662.0 cells/µL), *p* = 0.021; and the percentage of CD4+ lymphocytes was 33.8% (IQR 29.4‐41.7%) vs. 42.4% (IQR 40.4–48.6%), *p* = 0.000. Furthermore, serum ferritin levels exhibited a linear positive correlation with CRP (*r* = 0.420, *p* = 0.001) and a negative correlation with CD4+ lymphocyte count (*r* = −0.412, *p* = 0.007) and percentage of CD4+ lymphocytes (*r* = −0.567, *p* = 0.000).

**Conclusions:**

Patients with KFD demonstrated immunosuppression characterized by decreased counts of circulating CD3+ and CD4+ lymphocytes, as well as NK cells. Moreover, serum ferritin levels were inversely correlated with CD4+ lymphocyte counts, suggesting a potential role of ferritin in immune dysregulation in KFD.

## Graphical abstract

This retrospective cohort study indicates that patients with Kikuchi–Fujimoto disease demonstrated immunosuppression characterized by decreased counts of circulating CD3+ and CD4+ lymphocytes. Moreover, serum ferritin levels were inversely correlated with CD4+ lymphocyte counts, suggesting a potential role of ferritin in immune dysregulation in Kikuchi–Fujimoto disease.

## 1 Introduction

Kikuchi–Fujimoto disease (KFD), also known as histiocytic necrotizing lymphadenitis, is a rare non‐cancerous lymph node disorder characterized by fever and swollen, painful lymph nodes, which can persist for several weeks. This disease manifests as self‐limited nonsuppurative inflammation and is prone to misdiagnosis as lymphoma, adult Steele's disease, tuberculosis, hemophagocytic lymphohistiocytosis (HLH), or Kawasaki disease. Accurate diagnosis often necessitates histopathological examination of lymph nodes [1].

The etiology and pathogenesis of KFD remain unclear, but they are believed to involve a robust lymphocyte‐mediated immune response triggered by various antigens in genetically susceptible populations. Tanaka et al. observed significantly higher frequencies of DPA101 and DPB10202 alleles in HLA class II genes among patients with KFD compared to those without. DPB10202 alleles are relatively common in Asian populations, likely contributing to the relatively high incidence of KFD in Asia [2]. Additionally, infections with Epstein‐Barr virus (EBV), cytomegalovirus (CMV), varicella‐zoster virus (VZV), adenovirus, and parvovirus B19 may induce abnormal lymphocyte responses to local or systemic stimuli in patients with KFD [1, 3, 4]. EBV has been proven to be related to the pathogenesis of autoimmune diseases such as systemic lupus erythematosus (SLE) and Sjogren's syndrome. Meanwhile, KFD and autoimmune diseases also have common clinical and laboratory manifestations. Most studies contend that the association between them cannot be purely attributed to chance [5].

Previous studies have indicated that serum ferritin, an acute inflammatory reactant, increases in KFD and can inhibit lymphocyte proliferation [1, 6, 7, 8, 9]. However, the relationship between serum ferritin and lymphocyte subsets in the peripheral blood of patients with KFD has not been elucidated. Therefore, we conducted a retrospective study to investigate this aspect and provide insights into the immune response in KFD.

## 2 Methods

### 2.1 Study Participants and Their Evaluation

**Figure 1:**
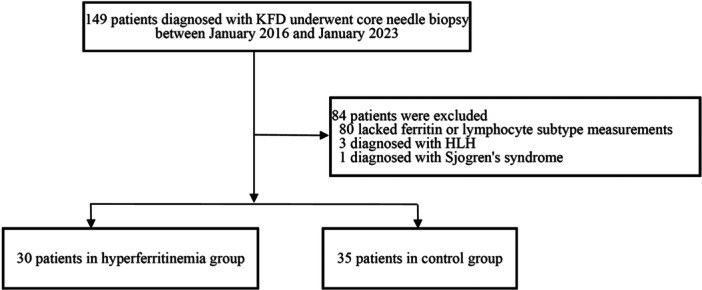
Inclusion flow chart.

This study received approval from the Ethics Committee of Beijing Friendship Hospital, Capital Medical University in China with informed consent exemption (reference number 2023PHB166‐001). The reporting of this study conforms to the Strengthening the Reporting of Observational Studies in Epidemiology (STROBE) statement: guidelines for reporting observational studies [10]. Between January 2016 and January 2023, 149 patients diagnosed with KFD underwent ultrasound‐guided core needle biopsy at Beijing Friendship Hospital and were subsequently admitted for treatment. Among them, 84 patients were excluded from our study: 3 patients were diagnosed with HLH, 1 patient was diagnosed with Sjogren's syndrome, and 80 patients lacked serum ferritin measurements. Clinical data were collected and analyzed from the remaining 65 patients. The normal serum ferritin concentration ranges from 24 to 336 μg/L for males and 11 to 306 μg/L for females. The normal reference range for serum ferritin concentration was obtained from measurements in 200 healthy males and 200 healthy females. Due to its skewed distribution, the reference range was calculated using the percentile method (P_5_‐P_95_). Patients were categorized into two groups based on serum ferritin levels, with 30 patients in the hyperferritinemia group and 35 patients in the control group (Figure 1). Prior to admission, all patients had received treatment with nonsteroidal anti‐inflammatory drugs, traditional Chinese medicine, and antibiotics (including cephalosporins, azithromycin, and quinolones), while 6 patients had received short‐term intravenous glucocorticoids.

This retrospective cohort study collected baseline characteristics, medical histories, laboratory test results, lymph node ultrasound examination findings, and lymph node pathology results from an electronic medical record system for analysis. This study did not conduct a follow‐up on the recurrence of KFD. The onset date was defined as the date of initial symptom appearance. Laboratory tests included routine blood work, blood biochemistry, serum ferritin, C‐reactive protein (CRP), erythrocyte sedimentation rate (ESR), lymphocyte subset determination, and virus detection. serum ferritin concentrations were measured with a Roche Cobas e601 clinical analyzer (Roche Diagnostics, Indianapolis, USA). lymphocyte subtypes were analyzed by flow cytometry with a Becton Dickinson FACSCanto II cell analyzer (Becton Dickinson, Franklin Lakes, NJ, USA)

KFD is challenging to differentiate from other lymph node lesions solely through hematoxylin‐eosin staining (HE) of lymph node tissue. Therefore, a combination of immunohistochemical staining was utilized for differential diagnosis, encompassing CD3, CD5, CD7, CD20, CD21, CD30, CD56, CD68, CD123, CD163, MPO, Ki‐67, EBER, BCL‐2, and BCL‐6. The immunohistological landscape of KFD is characterised by increased numbers of plasmacytoid dendritic cells that frequently cluster around apoptotic/necrotic foci, increased numbers of cytotoxic T cells, and substantial distortion of follicular dendritic cell meshworks [4]. All pathological diagnoses were confirmed by at least two pathologists.

Given the controversy surrounding whether viral infection is the etiology of KFD, only a subset of patients in our retrospectively collected cases underwent virus screening. EBV was detected in plasma or peripheral blood mononuclear cells (PBMC) using nucleic acid probes, alongside the detection of human anti‐EBV IgM (EBV‐IgM), anti‐EBV capsid antigen IgG (EBVCA‐IgG), anti‐EBV early antigen IgG (EA‐IgG), and/or anti‐EBV nuclear antibody IgG (EBNA‐IgG) antibodies via enzyme‐linked immunosorbent assays. Polymerase chain reaction was employed to detect CMV DNA in blood. Furthermore, some patients were tested for IgM antibodies against cytomegalovirus (CMV‐IgM), adenovirus (AdV‐IgM), herpes simplex virus type 1 (HSV1‐IgM), herpes simplex virus type 2 (HSV2‐IgM), rubella virus (RBV‐IgM), group B Coxsackievirus (COxB‐IgM), and respiratory syncytial virus (RSV‐IgM).

### 2.2 Statistical Analyses

Categorical variables were reported as frequencies and percentages, while continuous variables were described as medians with interquartile ranges (IQR). One‐way analysis of variance (ANOVA) determined group differences for continuous variables adhering to a normal distribution, while non‐parametric tests were utilized for non‐normally distributed continuous variables. The *χ* ^2^ test assessed associations among categorical variables, and Pearson correlation analysis computed correlation coefficients. A significance level of *p* < 0.05 indicated statistical significance. Statistical analyses were conducted using SPSS version 24.0 (IBM, Armonk, NY, United States).

## 3 Results

Patients ranged in age from 9 to 48 years, with a median age of 27.0 years (IQR 22.0–32.0 years) across all patients. The median ages of the hyperferritinemia and control groups were 31.0 years (IQR 26.5–37.0 years) and 24.5 years (IQR 22.0–29.5 years), respectively, with a significant difference noted (*p* = 0.048).

Of the total 65 patients, 30 were male and 35 were female. The proportion of females in the hyperferritinemia group was 43.3%, while in the control group, it was 62.9%. No statistically significant difference in sex distribution was observed between the two groups (*p* = 0.140). The duration from symptom onset to admission for all patients was a median of 20.0 days (IQR 14.0–30.0 days), with comparable durations noted for the hyperferritinemia group (20.0 days, IQR 14.0–30.0 days) and the control group (25.0 days, IQR 14.0–30.0 days) (*p* = 0.130). Among all patients, 9 (13.8%) had comorbidities such as diabetes mellitus, hypertension, hyperuricemia, rheumatoid arthritis, polycystic ovarian syndrome, Hashimoto's thyroiditis, and neurodermatitis, but no disease was predominant. There was no statistically significant difference in comorbidity prevalence between the hyperferritinemia and control groups (*p* = 0.282) (Table 1).

**Table 1:** Demographics and baseline characteristics of patients with KFD.

	No. (%) Total (*n* = 65)	Hyperferinemia (*n* = 30)	Control (*n* = 35)	*p*‐value
Age in years, median (IQR)	27.0 (22.0–32.0)	31.0 (26.5–37.0)	24.5 (22.0–29.5)	0.048
Sex				
Male	30 (46.2)	17 (56.7)	13 (37.1)	0.140
Female	35 (53.8)	13 (43.3)	22 (62.9)	
Duration of onset before admission, median (IQR)	20.0 (14.0–30.0)	20.0 (14.0–30.0)	25.0 (14.0–30.0)	0.130
Comorbidities	9 (13.8)	6 (20.0)	3 (8.6)	0.282
Diabetes mellitus	1 (1.5)	1 (3.3)	0	
Hypertension	3 (4.6)	2 (6.7)	1 (2.9)	
Hyperuricemia	1 (1.5)	1 (3.3)	0	
Rheumatoid arthritis	1 (1.5)	1 (3.3)	0	
Polycystic ovary syndrome	1 (1.5)	1 (3.3)	0	
Hashimoto's thyroiditis	1 (1.5)	0	1 (2.9)	
Neurodermatitis	1 (1.5)	0	1 (2.9)	

Abbreviation: IQR, interquartile range.

The biopsied lymph nodes were predominantly located in the neck (89.2%), with fewer occurrences in the axilla (6.2%) and submandible (4.6%). The median long diameter of the biopsied lymph nodes was 2.3 mm (IQR 2.0–2.8 mm), while the short diameter was 0.9 mm (IQR 0.7–1.2 mm). Comparison between the hyperferritinemia and control groups revealed similar measurements for the long diameter: 2.2 mm (IQR 1.8–2.5 mm) and 2.3 mm (IQR 2.0–3.1 mm), respectively, with no statistically significant difference noted (*p* = 0.195). Similarly, the short diameter measurements were comparable between the groups: 0.8 mm (IQR 0.7–1.0 mm) for the hyperferritinemia group and 0.9 mm (IQR 0.7–1.2 mm) for the control group, with no significant difference observed (*p* = 0.419) (Table 2).

**Table 2:** Comparison of ultrasound images of lymph nodes in patients with KFD.

	No. (%) Total (*n* = 65)	Hyperferinemia (*n* = 30)	Control (*n* = 35)	*p*‐value
Location				
Neck	58 (89.2)	27 (90.0)	31 (88.6)	
Axillae	4 (6.2)	2 (6.7)	2 (5.7)	
Submandibular	3 (4.6)	1 (3.3)	2 (5.7)	
Size (IQR)				
Long diameter (cm)	2.3 (2.0–2.8)	2.2 (1.8–2.5)	2.3 (2.0–3.1)	0.195
Short diameter (cm)	0.9 (0.7–1.2)	0.8 (0.7–1.0)	0.9 (0.7–1.2)	0.419

Abbreviation: IQR, interquartile range.

Out of 51 patients who underwent plasma EBV‐DNA testing, only 1 patient (2.0%) from the hyperferritinemia group tested positive, while none in the control group were positive. Among the 23 patients who underwent PBMC EBV‐DNA testing, 3 (13.0%) from the control group were positive, while none in the hyperferritinemia group were positive. Among the 10 patients (33.3%) in the hyperferritinemia group who underwent viral antibody testing, all tested negative for EBV‐IgM, 9 (90.0%) tested positive for VCA‐IgG, 5 (50.0%) tested positive for EAI‐gG, and 8 (80.0%) tested positive for EBNA‐IgG. Similarly, in the control group, out of 6 patients (17.1%) tested for anti‐EBV antibodies, all tested negative for EBV‐IgM, but positive for VCA‐IgG and EBNA‐IgG. All individuals tested for CMV‐DNA, CMV‐IgM, and RSV‐IgM returned negative results (Table 3).

**Table 3:** Comparison of virus screening in patients with KFD.

	Total (*n* = 65)	High ferritin (*n* = 30)	Control (*n* = 35)
EBV			
EBV‐DNA(Plasma)	1	1	0
EBV‐DNA(PBMC)	3	0	3
EBV‐IgM	0	0	0
EBVCA‐IgG	15	9	6
EA‐IgG	7	5	2
EBNA‐‐IgG	14	8	6
CMV			
CMV‐DNA	0	0	0
CMV‐IgM	0	0	0
AdV‐IgM	2	2	0
HSV1‐IgM	2	1	1
HSV2‐IgM	2	1	1
RBV‐IgM	1	1	0
COxB‐IgM	1	1	0
RSV‐IgM	0	0	0

Abbreviations: AdV‐IgM, adenovirus IgM; CMV, cytomegalo virus; COxB‐IgM, Coxsackie B group virus IgM; EA‐IgG, epstein‐barr virus early antigen IgG; EBNA‐IgG, epstein‐barr virus nuclear antibody IgG; EBV, epstein‐barr virus; EBVCA‐IgG, epstein‐barr virus capsid antigen IgG; HSV1‐IgM, herpes simplex virus type 1 IgM; HSV2‐IgM, herpes simplex virus type 2 IgM; IQR, interquartile range; PBMC, peripheral blood mononuclear cell; RBV‐IgM, rubella virus IgM; RSV‐IgM, respiratory syncytial virus IgM.

The serum ferritin levels of all patients were 289.4 µg/L (IQR 108.0–653.1 µg/L), with the hyperferritinemia group and control group exhibiting levels of 664.4 µg/L (IQR 450.5–963.9 µg/L) and 111.3 µg/L (IQR 75.7–207.4 µg/L), respectively. Both groups exceeded the normal range for CRP, with levels of 25.5 mg/L (IQR 16.4–54.4 mg/L) vs. 20.0 mg/L (IQR 11.7–35.9 mg/L), respectively (*p* = 0.121); ESR, with levels of 33.5 mm/h (IQR 22.8–51.5 mm/h) vs. 25.0 mm/h (IQR 18.0–37.0 mm/h), respectively (*p* = 0.245); and LDH, with levels of 508.0 µg/L (IQR 339.0–582.0 µg/L) vs. 334.5 µg/L (IQR 275.8–437.0 µg/L), respectively (*p* = 0.118) (Table 4).

**Table 4:** Comparison of laboratory parameters in patients with KFD.

	Median (IQR)				
	Normal range	Total (*n* = 65)	High ferritin (*n* = 30)	Control (*n* = 35)	*p*‐value
					
Serum ferritin (µg/L)		289.4 (108.0–653.1)	664.4 (450.5–963.9 8)	111.3 (75.65–207.35)	< 0.000
CRP (mg/L)	< 1.0	22.6 (12.4–48.8)	25.5 (16.4–54.4)	20.0 (11.7–35.9)	0.121
ESR (mm/H)	0.0–15.0	28.0 (19.0–41.5)	33.5 (22.8–51.5)	25.0 (18.0–37.0)	0.245
LDH (u/L)	135.0–225.0	397.0 (283.5–537.5)	508.0 (339.0–582.0)	334.5 (275.8–437.0)	0.118
Leukocytes (×10^9^/L)	3.5–9.5	2.8 (2.1–3.4)	2.8 (2.4–3.4)	2.7 (2.1–3.4)	0.797
NE (%)	40.0–75.0	45.4 (38.0–53.2)	50.9 (44.3–58.7)	42.5 (37.9–48.2)	0.058
LY (%)	20.0–50.0	43.0 (36.1–49.9)	40.9 (33.5–46.8)	43.9 (38.7–50.5)	0.299
MO (%)	3.0–10.0	9.9 (8.2–12.1)	9.6 (7.1–12.2)	10.0 (8.9–12.0)	0.475
Lymphocytes (×10^9^/L)	1.1–3.2	1.1 (0.82–1.4)	1.2 (0.7–1.3)	1.0 (0.9–1.4)	0.669
CD3 + (/µL)	955.0–2860.0	890.8 (656.5–1105.0)	945.0 (554.4–1087.8)	815.4 (677.3–1104.9)	0.432
CD3 + (%)	50.0–84.0	78.2 (74.9–83.4)	77.9 (74.8–83.1)	79.9 (75.9–83.4)	0.487
CD4 + (/µL)	550.0–1440.0	403.5 (300.3–559.9)	387.7 (261.5–479.7)	479.9 (396.2–662.0)	0.021
CD4 + (%)	27.0–51.0	40.2 (32.5–45.0)	33.8 (29.4–41.7)	42.4 (40.4–48.6)	< 0.000
CD8 + (/µL)	320.0–1250.0	367.4 (269.1–553.5)	473.3 (259.3–561.2)	338.6 (288.0–476.0)	0.535
CD8 + (%)	15.0–44.0	36.3 (32.1–39.9)	38.6 (35.0–43.9)	34.0 (30.0–36.7)	0.010
CD19 + (/µL)	90.0–560.0	115.1 (66.6–157.4)	115.1 (51.2–159.9)	119.5 (84.4–151.4)	0.973
CD19 + (%)	5.0–18.0	10.6 (7.6–13.4)	9.9 (6.6–13.0)	11.3 (7.9–14.0)	0.561
NK (/µL)	150.0–1100.0	80.2 (50.7–129.7)	104.0 (52.4–138.1)	67.2 (45.1–110.1)	0.281
NK (%)	7.0–40.0	8.5 (5.3–13.0)	9.9 (7.2–13.6)	5.7 (4.5–11.9)	0.295

Abbreviations: ESR, erythrocyte sedimen; lCRP, C‐reactive protein; IQR, interquartile range.

The leukocyte count of all patients decreased to 2.8 × 109/L (IQR 2.1–3.4), with no significant difference between the two groups (*p* = 0.797). The counts of CD3+ lymphocytes, CD4+ lymphocytes, and NK cells were all low: CD3+ lymphocytes, 945.0 cells/µL (IQR 554.4–1087.8 cells/µL) vs. 815.4 cells/µL (IQR 677.3–1104.9 cells/µL), respectively (*p* = 0.432); CD4+ lymphocytes, 387.7 cells/µL (IQR 261.5–479.7 cells/µL) vs. 479.9 cells/µL (IQR 396.2–662.0 cells/µL), respectively (*p* = 0.021); NK cells, 104.0 cells/µL (IQR 52.4–138.1 cells/µL) vs. 67.2 cells/µL (IQR 45.1.3–110.1 cells/µL), respectively (*p* = 0.281). The percentage of CD4+ lymphocytes in total lymphocytes between the two groups was 33.8% (IQR 29.4–41.7%) vs. 42.4% (IQR 40.4–48.6%), respectively (*p* = 0.000). There was no significant change in CD8+ lymphocyte or CD19+ lymphocyte counts between the two groups (Table 4).

**Figure 2:**
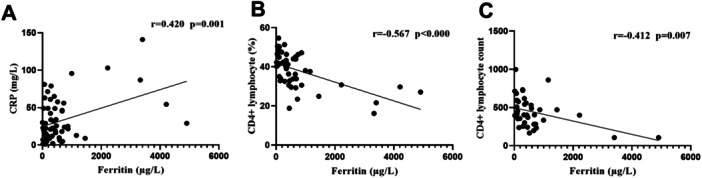
Correlation analysis of C‐reactive protein, CD4+ lymphocytes, and serum ferritin level. (A) a linear positive correlation between serum ferritin and CRP in all patients; (B and C) a negative correlation between serum ferritin and CD4+ lymphocyte percentage and CD4+ lymphocyte count.

**Figure 3:**
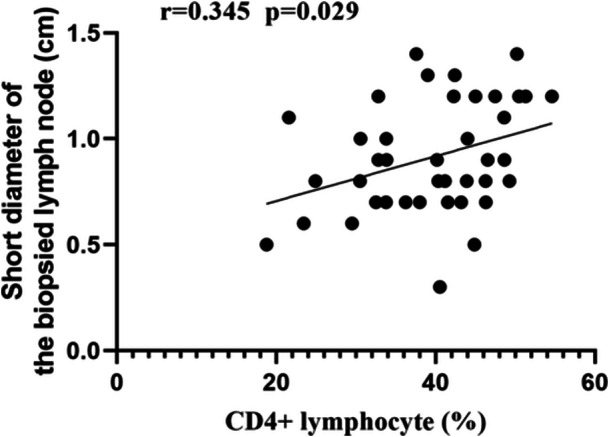
Correlation analysis between percentage of CD4+ lymphocytes and short diameter of biopsied lymph nodes.

Pearson correlation analysis demonstrated a linear positive correlation between serum ferritin in all patients and CRP (*r* = 0.420, *p* = 0.001), and a negative correlation with CD4+ lymphocyte percentage (*r* = −0.567, *p* = 0.000), and CD4+ lymphocyte count (*r* = −0.412, *p* = 0.007). Furthermore, the percentage of CD4+ lymphocytes weakly correlated positively with the short diameter of lymph nodes (*r* = 0.345, *p*= 0.029) (Figures 2 and 3).

## 4 Discussion

KFD, characterized by fever and swollen, painful lymph nodes commonly found in the neck, predominantly affects young adults without specific comorbidities, and its viral etiology remains unconfirmed [1, 3, 9, 11]. While lymph node ultrasound examination lacks specific diagnostic value, core needle biopsy, particularly with immunohistochemical staining, plays a crucial role in distinguishing KFD from other diseases such as lymphadenitis and lymphoma [1, 4, 12, 13].

KFD manifests in three morphological phases: proliferative, necrotizing, and xanthomatous, depending on the presence or absence of necrosis and lipid‐laden histiocytes. Immunohistochemical analysis reveals the presence of Fas and FasL in histiocytes and lymphocytes in KFD, with CD4+ lymphocytes predominating in the early stages and CD8+ lymphocytes prevailing in the more common stages of necrosis [4, 11, 14]. This underscores a robust immune response involving lymphocytes within KFD lymph nodes. Building upon these findings, our study further delineates changes in the number and composition of peripheral blood lymphocytes in KFD patients: although no significant alterations were noted in the total count of lymphocytes, CD8+ lymphocytes, or CD19+ lymphocytes, there was a notable reduction in CD3+ lymphocytes, CD4+ lymphocytes, and NK cells. Intriguingly, we observed a negative correlation between CD4+ lymphocytes and serum ferritin levels.

Ferritin is widely recognized as a biomarker of acute inflammation and actively participates in the regulation of inflammatory responses [15, 16, 17, 18]. Present in the nucleus, mitochondria, lysosomes, and plasma, ferritin serves as a central mediator of iron homeostasis. By sequestering up to 4500 iron atoms, ferritin mitigates the concentration of divalent iron ions, thus curbing the generation of free radicals, consequently attenuating oxidative stress, safeguarding DNA and mitochondria from reactive oxygen species, impeding inflammatory reactions, and forestalling ferroptosis [19, 20]. The study by Weis et al. showed that adding exogenous ferritin improved the prognosis of sepsis [21]. Our study establishes a positive correlation between elevated serum ferritin levels and CRP in KFD patients, indicating serum ferritin as an additional inflammatory marker for this disease. Furthermore, based on previous reports, serum ferritin may confer protective effects in KFD pathogenesis.

Ferritin can inhibit the proliferation of lymphoid and myeloid cells, and may improve the prognosis of mice with sepsis through this immunosuppressive mechanism [6, 7, 8]. Fargion et al. confirmed that ferritin can inhibit lymphocytes activated by plant hemagglutinin and concanavalin A, and identified binding sites for ferritin on the surface of human lymphocytes [22]. Ferritin binding to T cell immunoglobulin and mucin‐containing molecule 2 (TIM‐2) impedes lymphocyte activation and proliferation [8, 23, 24], modulates subpopulation distribution, and inhibits CD4+ lymphocytes [17, 18, 25]. Additionally, ferritin‐stimulated regulatory T cells exhibit elevated interleukin‐10 (IL‐10) production, an anti‐inflammatory cytokine mediating immunosuppression, thus further impeding lymphocyte activity [6, 7, 26]. The findings demonstrate a negative correlation between CD4+ lymphocytes and serum ferritin levels in the peripheral blood of KFD patients, suggesting the potential for ferritin to directly and indirectly inhibit CD4+ lymphocyte proliferation through these mechanisms.

This study represents the first documentation of alterations in lymphocyte subtypes in the peripheral blood of KFD patients and the correlation between CD4+ lymphocytes and serum ferritin levels. Our findings offer insights into the immune response in KFD, although several limitations warrant consideration: (1) The rarity of KFD limits patient enrollment, thus caution is advised in interpreting results from this small retrospective study; (2) Our investigation solely focused on numerical changes rather than functional characteristics of peripheral blood lymphocyte subtypes; (3) While ferritinemia serves as an indicator of macrophage activation in HLH, its correlation with macrophage activation in KFD remains unexplored due to the lack of relevant data in our retrospective study. Additionally, naive CD4+ lymphocytes differentiate into one of several lineages, with hallmark transcription factors, cytokine production, and functions in vivo, according to the particular cytokine milieu. Further research is imperative to explore the correlation between serum ferritin and these lineages.

## 5 Conclusion

Patients with KFD demonstrated immunosuppression characterized by decreased counts of circulating CD3+ and CD4+ lymphocytes, as well as NK cells. Moreover, serum ferritin levels were inversely correlated with CD4+ lymphocyte counts, suggesting a potential role of ferritin in immune dysregulation in KFD.

## Author Contributions

**Peng Zhong:** data curation, formal analysis, methodology, project administration, writing – original draft. **Xiwen Sang:** conceptualization, data curation, formal analysis, resources. **Yu Yang:** data curation and resources. **Zhenzhou Wang:** conceptualization, data curation, formal analysis, funding acquisition, project administration, writing – original draft, writing – review and editing.

## Ethics Statement

This study received approval from the Ethics Committee of Beijing Friendship Hospital, Capital Medical University in China (reference number 2023PHB166‐001). Because only the medical records were reviewed, this case series was exempted from signing the informed consent.

## Conflicts of Interest

The authors declare no conflicts of interest.

## Data Availability

The data that support the findings of this study are available from the corresponding author, zhenzhou wang, upon reasonable request.
